# Atrial fibrillation is associated with increased central blood pressure and arterial stiffness

**DOI:** 10.1111/jch.14323

**Published:** 2021-07-12

**Authors:** Priit Pauklin, Jaan Eha, Kaspar Tootsi, Rein Kolk, Rain Paju, Mart Kals, Priit Kampus

**Affiliations:** ^1^ Department of Cardiology Institute of Clinical Medicine University of Tartu Tartu Estonia; ^2^ Heart Clinic Tartu University Hospital Tartu Estonia; ^3^ Department of Traumatology and Orthopedics Institute of Clinical Medicine University of Tartu Tartu Estonia; ^4^ Estonian Genome Center Institute of Genomics University of Tartu Tartu Estonia; ^5^ Institute for Molecular Medicine Finland FIMM HiLIFE University of Helsinki Helsinki Finland; ^6^ Centre of Cardiology North Estonia Medical Centre Tallinn Estonia

**Keywords:** arterial stiffness, atrial fibrillation, beta blockers, central blood pressure, pulse wave velocity

## Abstract

Atrial fibrillation (AF) is the most common arrhythmia in clinical practice and beta blockers (BBs) are the drugs of choice for rate or rhythm control in these patients. The purpose of this study was to describe differences in arterial stiffness (AS), central blood pressure (cBP), and the role of BBs on cBP in patients with AF compared to healthy individuals. The authors included 76 patients with paroxysmal/persistent AF. Carotid‐femoral pulse wave velocity (PWV) and cBP were measured and compared with data from 75 healthy individuals. Patients with AF had higher PWV (8.0 m/s vs. 7.2 m/s, *p* < .001), central systolic blood pressure (cSBP) (118 mm Hg vs. 114 mm Hg, *p* = .033), central pulse pressure (cPP) (39 mm Hg vs. 37 mm Hg, *p* = .035) and lower pulse pressure amplification (PPA) (1.24 vs. 1.30, *p* = .015), without differences in peripheral blood pressure (pBP) and heart rate (HR). AF patients had significantly increased PWV (β= 0.500, *p* = .010, adjusted R² = 0.37) after adjustment for confounding factors. The use of BBs significantly reduced PPA (β = ‐0.059, *p* = .017, adjusted R² = 0.30). AF patients have higher PWV, cSBP, cPP, and lower PPA, compared to healthy patients. These findings support the role of AS in the development of AF. Use of BBs is related to a potential adverse effect on cBP.

## 1 INTRODUCTION

**FIGURE 1:**
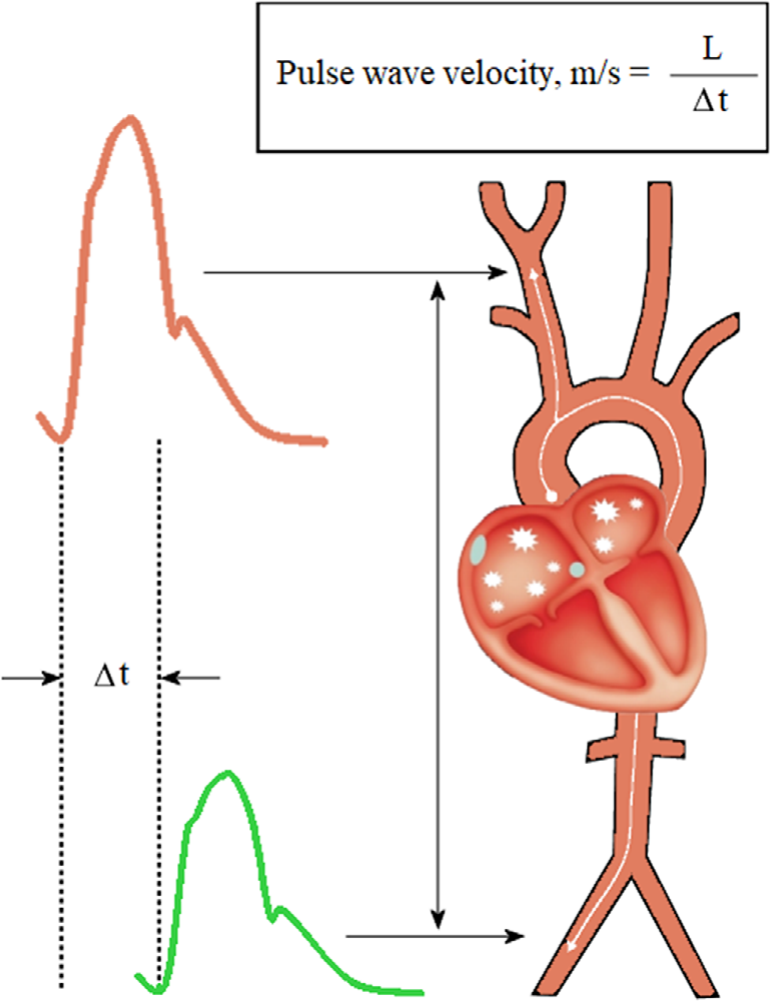
Principles of assessment of carotid‐femoral pulse wave velocity

Atrial fibrillation (AF) is the most common arrhythmia in clinical practice and remains one of the major causes of stroke, heart failure, sudden death, and cardiovascular morbidity in the world.^1^ Hypertension is the leading cardiovascular risk factor in the pathogenesis of AF. Due to the pulse pressure amplification (PPA) phenomenon occurring across the arterial tree, blood pressure (BP) and pulse pressure (PP) are known to be higher when assessed at the brachial artery (peripheral blood pressure–pBP) compared to the aorta (central blood pressure–cBP).^2^ Arterial stiffness (AS) and cBP are increasingly recognized important risk factors for cardiovascular disease,^3^, ^4^, ^5^, ^6^ but they have rarely been studied in AF patients (Figure 1).

Beta blockers (BBs) are the first‐choice drugs for long‐term rate and initial rhythm control for patients with AF. On the other hand, they are used less often for patients with hypertension because of their unfavorable effects on cBP compared to other antihypertensive medications.^7^, ^8^ Existing recommendations are somewhat contradictory, considering that hypertension is the most common risk factor for AF.^9^ Also, there is a lack of knowledge about the effect of BBs on the central hemodynamics of patients with AF. The aim of this study was to assess differences in the hemodynamic profile between patients with paroxysmal or persistent AF and a control group and to assess the role of BBs on cBP.

## 2 METHODS

### 2.1 Study population

The study population consisted of 27 patients with persistent AF, who were hospitalized for cardioversion, and 49 patients with paroxysmal AF who were hospitalized for pulmonary vein isolation (PVI) to the Department of Cardiology, Tartu University Hospital, and the Centre of Cardiology, North Estonia Medical Centre, Estonia, respectively.

The inclusion criteria were: age 18–75 years, successful restoration of sinus rhythm (SR) after cardioversion or SR before PVI procedure.

We excluded patients with contraindications for cardioversion, unsuccessful cardioversion, any acute or chronic inflammatory disease, known moderate to severe valve pathology, heart failure and malignancy

Age matched control patients with no history of AF or any other arrhythmia were recruited from family doctor's practices. We excluded patients with acute or chronic inflammatory disease, heart failure, known valve pathology and malignancy.

The study protocol was approved by the Research Ethics Committee of the University of Tartu. The study was conducted in accordance with the Declaration of Helsinki and written consent was obtained from each participant.

### 2.2 Study protocol

Blood samples were collected from the antecubital fossa after an overnight fast. BP and carotid‐femoral pulse wave velocities (PWV) were measured and pulse wave analysis (PWA) was performed within 1 day in patients after successful restoration of SR with cardioversion. Measurements of BP, PWV, and PWA were made before the procedure in patients who were hospitalized for PVI. All measurements were performed after 15 min of rest in a quiet, temperature‐controlled room in a supine position. All patients were in SR during the study.

### 2.3 Hemodynamic measurements

BP was measured, using a validated digital oscillometric device (A&D UA‐767; A&D Company Ltd., Tokyo, Japan), at least twice and mean BP was recorded.

Carotid‐femoral PWV was measured and PWA was performed, using a Sphygmocor device (Sphygmocor Xcel and Sphygmocor Px, AtCor Medical, Sydney, Australia), at least twice and mean values were recorded.

The quality of measurements for PWA and PWV were controlled using the Sphygmocor Xcel's build in quality control (QC) indicator. All measurements that did not meet the QC requirements (at least peripheral waveform quality above or equal to 75%) were dismissed and repeated.

PPA was calculated as a ratio of peripheral pulse pressure to central pulse pressure (pPP/cPP).

All measurements were performed in a dedicated, temperature controlled, study room for both the study, and control patients.

### 2.4 Echocardiography

Echocardiography was done as part of the clinical management of the patients elected for cardioversion or PVI in the study group. Echocardiographic information was available for all patients of the study group (76) and for 29 patients of the control group. The investigations were performed by experienced personnel.

### 2.5 Laboratory analysis

C‐reactive protein, creatinine, estimated glomerular filtration rate (eGFR), plasma glucose levels, complete blood count, total cholesterol, LDL‐cholesterol, HDL‐cholesterol, and triglycerides were measured by standard laboratory methods in the local clinical laboratory.

### 2.6 Statistical analysis

The statistical programs Statistica^10^ and R^11^ were used for statistical analysis. AF and control groups were compared using the Student's t‐test on continuous variables and the chi‐square test on categorical variables. The Pearson's correlation was used to evaluate linear relationships between continuous variables. A *p* value of < .05 was considered statistically significant.

Multiple linear regression analyses were performed to investigate the associations of carotid‐femoral PWV and PPA on a set of predictors. Both models were adjusted for potential confounders and samples containing any missing values were excluded. The confounders for the adjustment were chosen as follows: a t‐test was performed to see major differences between the control and study group. A stepwise approach was then used to add the confounders to the multivariate analysis. If the covariate did not contribute to the model's predictive value and was not statistically important, then it was removed. Some well‐known important confounders were shown in the analysis despite not being significant.

## 3 RESULTS

The study population consisted of 76 patients with AF and 75 age matched controls. The general characteristics of the AF patients and the control patients are described in Table 1.

**TABLE 1:** Baseline clinical characteristics of the study group and the control group

Variable	AF group (*n* = 76)	Control group (*n* = 75)	*p* value
Age (years)	57 (±9)	54 (±11)	.064
Male sex (*n* (%))	55 (72)	38 (51)	.010
Height (cm)	175 (±10)	172 (±10)	.778
Weight (kg)	90.2 (±16.5)	76.3 (±16.1)	<.001
Body mass index (kg/m^2^)	29.4 (±4.7)	25.3 (±4.7)	<.001
Peripheral systolic blood pressure (mm Hg)	127 (±13)	123 (±13)	.131
Peripheral diastolic blood pressure (mm Hg)	78 (±9)	76 (±8)	.142
Peripheral pulse pressure (mm Hg)	48 (±9)	47 (±9)	.365
Central systolic blood pressure (mm Hg)	118 (±14)	114 (±12)	.033
Central diastolic blood pressure (mm Hg)	79 (±9)	77 (±8)	.179
Central pulse pressure (mm Hg)	39 (±9)	37 (±8)	.035
Central mean arterial pressure (mm Hg)	94 (±10)	91 (±9)	.101
Pulse pressure amplification	1.24 (±0.14)	1.30 (±0.13)	.015
Systolic blood pressure amplification	1.07 (±0.04)	1.09 (±0.04)	.039
Heart rate (beats per minute)	58 (±9)	61 (±7)	.076
Augmentation pressure (mm Hg)	12 (±6)	9 (±5)	.001
Augmentation index (%)	29 (±11)	23 (±12)	.006
Augmentation index at heart rate of 75 beats per minute (%)	21 (±11)	18 (±13)	.085
Pulse wave velocity (m/s)	8.0 (±1.2)	7.2 (±1.2)	<.001
Diagnosis of hypertension (*n* (%))	49 (65)	2 (3)	<.001
Use of beta blockers (*n* (%)), metoprolol (*n* (%)), bisoprolol (*n* (%)), nebivolol (*n* (%))	68 (90) 61 (90) 4 (6) 3 (4)	0 (0)	<.001
Use of ACEIs, ARBs (*n* (%))	41 (54)	1 (1)	<.001
Estimated glomerular filtration rate (ml/min/1.73 m^2^)	83 (±15)	88 (±13)	.037
Creatinine (μmol/L)	84 (19)	75 (13)	.001
C‐reactive protein (mg/L)	2.47 (±3.52)	1.08 (±1.21)	.003
Total cholesterol (mmol/L)	5.46 (±1.10)	5.39 (±1.06)	.700
HDL‐cholesterol (mmol/L)	1.41 (±0.46)	1.67 (±0.45)	<.001
LDL‐cholesterol (mmol/L)	3.70 (±1.04)	3.68 (±0.79)	.866
Triglycerides (mmol/L)	1.52 (±0.70)	1.34 (±0.75)	.145

Values are presented as mean ± SD or count (%).

*Abbreviations*: SD, standard deviation; ACEIs, angiotensin‐converting enzyme inhibitors; ARBs, angiotensin II receptor blockers.

### 3.1 Hemodynamics

Patients with a history of AF had higher PWV (8.0 m/s vs. 7.2 m/s, *p* < .001), central systolic blood pressure (cSBP) (118 mm Hg vs. 114 mm Hg, *p* = .033), central pulse pressure (cPP) (39 mm Hg vs. 37 mm Hg, *p* = .035) and lower PPA (1.24 vs. 1.30, *p* = .015) compared to the control group, without differences in peripheral systolic pressure (pSBP) (127 mm Hg vs. 123 mm Hg, *p* = .131) and peripheral pulse pressure (pPP) (48 mm Hg vs. 47 mm Hg, *p* = .365). There was no difference in heart rate (HR) (58 vs. 61 bpm, *p* = .076).

Multiple linear regression analysis with carotid‐femoral PWV as a dependent variable (adjusted R² = 0.37), where HR, weight, mean central arterial pressure, glomerular filtration rate, and group status were considered as predictors, indicates that AF patients have significantly increased carotid‐femoral PWV (β = 0.500, *p* = .010). (Table 2)

**TABLE 2:** Multivariate linear regression analysis (adjusted R² = 0.37) using carotid‐femoral pulse wave velocity as the dependent variable (*n* = 148)

Variables	Beta	SE of beta	*p* value
Sex: female	0.007	0.006	.291
Age	0.070	0.009	<.001
Weight	‐0.227	0.202	.263
cMAP	0.019	0.009	.040
Heart rate	‐0.006	0.011	.552
eGFR	0.019	0.007	.008
Group: AF	0.500	0.193	.010

*Abbreviations*: SE, standard error; cMAP, central mean arterial pressure; eGFR, estimated glomerular filtration rate.

PPA was significantly lower in patients who took BBs compared to patients who did not take BBs (1.25 vs. 1.30, *p* = .037). In a multiple linear regression analysis with PPA as a dependent variable (adjusted R² = 0.30), where body mass index, diagnosis of hypertension, use of angiotensin‐converting enzyme inhibitors (AKEIs) or angiotensin receptor blockers (ARBs), and use of BBs were considered as independent variables, the use of BBs was associated with significantly lower PPA (β = ‐0.059, *p* = .017). At the same time, AKEI/ARB use did not have a significant effect on PPA (β = 0.044, *p* = .243). (Table 3)

**TABLE 3:** Multivariate linear regression analysis (adjusted R² = 0.30) using pulse pressure amplification as the dependent variable (*n* = 150)

Effect	Beta	SE of beta	*p* value
Age	‐0.007	0.001	<.001
Sex: female	‐0.067	0.021	.002
BMI	‐0.004	0.002	.087
Diagnosis of hypertension: yes	0.015	0.037	.677
Use of AKEIs/ARBs: yes	0.044	0.037	.243
Use of BB: yes	‐0.059	0.024	.017

*Abbreviations*: SE, standard error; BMI, body mass index; ACEIs, angiotensin‐converting enzyme inhibitors; ARBs, angiotensin II receptor blockers; BB, beta blockers.

### 3.2 Echocardiography

Patients with AF had mild dilatation of the left atria (LA) compared to the control group (23.1 ml/m^2^ vs. 36.4 ml/m^2^, *p* < .001). Analyzing the AF patients and controls together revealed a positive correlation of LA diameter (r = 0.38, *p* < .001) and indexed LA volume (r = 0.33, *p* = .001) with PWV. No statistically significant correlations were observed when the patients of the AF group and the controls were analyzed separately.

There was also a positive correlation of LA diameter with pSBP (r = 0.56, *p* = .002), cSBP (r = 0.51, *p* = .005), pPP (r = 0.42, *p* = .024) and cPP (r = 0.38, *p* = .043) in the control group. No significant correlation between BP and LA diameter was seen in the AF group. The echocardiography data is presented in Table 4.

**TABLE 4:** Echocardiographic measurements of patients with atrial fibrillation and control group

Variable	AF group (*n* = 76)	Control group (*n* = 29)	*p* value
Patients with atrial fibrillation during echocardiography (n (%))	28 (37)	0 (0)	<.001
Ejection fraction (%)	58.4 (±9.3)	65.5 (±5.1)	.001
Interventricular diastolic septum thickness (cm)	1.0 (±0.2)	0.8 (±0.1)	<.001
Left ventricular internal diastolic diameter (cm)	4.9 (±0.5)	4.9 (±0.6)	.368
Posterior wall diastolic thickness (cm)	1.0 (±0.2)	0.8 (±0.1)	.008
Left atrial diameter (cm)	4.0 (±0.4)	3.5 (±0.4)	<.001
Left atrial end systolic volume (ml)	74.2 (±22.9)	44.4 (±13.0)	<.001
Left atrial end systolic volume index (ml/m^2^)	36.4 (±9.6)	23.1 (±5.1)	<.001
Tricuspid annular plane systolic excursion (cm)	2.1 (±0.4)	2,2 (±0.3)	.303
Body surface area (m^2^)	2.0 (±0.2)	1.9 (±0.3)	.007

Values are presented as mean ± SD or count (%).

*Avbbreviation*: SD, standard deviation.

## 4 DISCUSSION

In this study patients with AF had higher PWV, cSBP, cPP, and lower PPA compared to healthy controls, without differences in HR, pSBP, or pPP. Treatment with BBs was associated with lower PPA.

AS is a recognized marker of cardiovascular risk,^3^ while carotid‐femoral PWV measurement has been widely accepted as the gold standard for assessing AS.^4^ AS has been found to be an independent predictor of primary coronary events^12^ and stroke in hypertensive patients.^5^

The interactions between AF and AS are not fully understood and information about the importance of AS in association with AF is scarce and contradictory. An earlier study with 34 patients and 31 controls did not find any difference in PWV between patients with first episode of AF and healthy patients.^13^ The reasons for the differences from our study might be that the mean age of the participants was younger (49 years vs. 57 years in our study), the prevalence of hypertension was lower (39% vs. 65%) and the study excluded patients with LA diameter over 40 mm. These differences indicate that the patients of our study group were in a more advanced stage of the disease, which might explain the discrepancy between the results.

Another study by Kizilirmak and coworkers^14^ compared cBP and AS in patients with paroxysmal AF and in the control group. They found that patients with paroxysmal AF had higher cBP and increased PWV. Also, there was a significant difference in pBP (133/83 mm Hg, vs. 120/75 mm Hg, *p* < ,001) between the patients and the control group, which correlated with difference in cBP.^14^ In the present study, despite the absence of a difference in pBP, the AF group showed higher cSBP and cPP. The importance of assessing cSBP and cPP was demonstrated in the Strong Heart Study,^6^ where cBP proved to be a better predictor for cardiovascular events than pBP in participants without clinical cardiovascular disease at baseline. Similar results were confirmed in a meta‐analysis by Vlachopoulus and coworkers^3^ A reduction in cBP with antihypertensive drugs better predicts further cardiovascular events than pBP.^8^ These findings support the theory that, compared to pBP, cBP reflects better the loading conditions for the heart, brain, and other organs. The higher cBP in our study population compared to the control might indicate a higher residual cardiovascular risk irrespective of having normal brachial BP levels. This is also supported by increased AS in the study patients compared to the control.

Lamante and coworkers showed that PWV and PP, a surrogate marker for AS, is correlated with LA size in hypertensive patients without previous AF episodes.^15^ This was also confirmed by another study with 111 hypertensive patients^16^ and by a larger prospective, community‐based observational study, where peripheral PP was predictive of AF incidence.^17^ These results demonstrate that increased AS may cause atrial enlargement, a known risk factor for AF.^18^, ^19^, ^20^ AS influences cardiac remodeling and left ventricular geometry and has an important role in the diastolic function of the ventricle,^21^ all of which are considered major determinants of LA size and hence contribute to their relationship with AF.^22^ In our study patients with AF had a larger LA diameter, indexed LA end systolic volume, and increased AS, compared to the control group. We found a positive correlation between PWV and size and volume of LA when AF patients and controls were analyzed together; however, no significant correlation was seen in the AF group. The reason for this might be that all AF patients were managed using a rhythm control strategy, which is usually opted for patients with milder structural changes in the heart.

Hypertension is the most prevalent, independent, and potentially modifiable risk factor for AF.^9^ Also, 65% of our study patients had a diagnosis of hypertension. As the prevalence of AF and hypertension increase with age, it is common to see AF patients with concomitant hypertension.^9^ Most of our study patients (90%) took BBs for rate or initial rhythm control and BP management. The most frequently used BB in our study was metoprolol (90% of all BBs). One reason for the higher cPP and cSBP in the study group might be that BBs have a smaller effect on cBP than on pBP. The use of BBs for the treatment of hypertension has been criticized because of its unfavorable effect on the cBP. Data from a meta‐analysis published by Law and coworkers also indicate a slight inferiority of BBs in preventing stroke.^23^ The Conduit Artery Function Evaluation (CAFE) Study^8^ demonstrated a more pronounced effect of ACEIs, ARBs and calcium channel blockers (CCBs) on cBP reduction, compared to the cardio‐selective BB atenolol.^8^ This was also confirmed in a meta‐analysis by Manisty and Hughes.^24^ However, the inferior performance on cBP does not seem to be a class effect. Our recent study showed that the vasodilating BB nebivolol reduced cBP, cPP, and left ventricular wall thickness significantly more than metoprolol, with comparable reduction in pBP and HR.^25^ The superior effect of nebivolol compared to atenolol regarding cBP reduction was also reported by Dhakam and coworkers^26^ There is some evidence that, through reducing HR, non‐vasodilating BBs may be associated with augmentation of cBP, thereby reducing the lowering effects on cBP.^8^, ^25^ On the other hand, in our recent study, pacemaker patients with sick sinus syndrome who had lower HR (60 beats per minute) *versus* higher heart rate (90 beats per minute), did not show any increase in cBP.^27^ In addition, Teeäär and coworkers showed that atenolol's inferior ability to reduce central BP in an acute setting may be related to heart rate‐dependent and ‐independent mechanisms.^28^

The current AF and hypertension guidelines^1^, ^29^ recommend BBs as the first‐line drugs for rate or initial rhythm control for patients with AF. According to our study, BBs were significantly linked to lower PPA. These findings coincide with the results of the CAFE study^8^ and a meta‐analysis,^24^ where BBs had a smaller effect on cBP compared to other antihypertensive drugs. This further confirms the need to assess cBP in order to better manage the higher cardiovascular risk of AF patients. Our findings may affect the choice of BBs within the class for rate control and BP management in patients with AF, favoring vasodilating BBs or non‐dihydropyridine CCBs. A combination therapy for BBs with AKEIs or ARBs might better target cBP, thereby reducing the higher residual risk resulting from higher cBP.^30^

Studies with non‐dihydropyridine CCBs and vasodilating BBs for assessment of HR and cBP in patients with AF should be undertaken to better understand the different impact of the mentioned drugs on central hemodynamics.

Although the link between AF and AS would produce a novel readily measurable target for AF prevention, the most suitable pharmacologic therapy for AS reduction has not yet been established. Previous studies with hypertensive patients have reported a potential impact of AKEIs on AS, which is partially independent of BP.^30^, ^31^ At the same time, a meta‐analysis by Shahin and coworkers showed that ACEIs significantly reduced PWV in comparison to placebo, but not in comparison to other antihypertensive drugs.^32^ Similar results were confirmed in a recent meta‐analysis by Xiuli and coworkers^33^ In our study AKEIs and ARBs did not have any adverse impact on PPA or PWV (data not shown).

Current study has some limitations to be addressed. The peculiarity of our study was that all hemodynamic measurements were made in SR in both groups. This method should help to overcome the potential inaccuracies of measurements of the central hemodynamics caused by HR variability in AF.^4^ This would improve measurement accuracy, but may lead to different result in patients with persistent AF. One study has looked at the feasibility of measurement of PWV and cPP in patients with AF before and after cardioversion.^34^ They found a decrease in PWV and increase in cPP after cardioversion but after adjusting for changes in MAP and HR they concluded that measurements of PWV and cPP were reliable in patients with AF.

Because this is a cross‐sectional design study, no strong causal claims can be made. We also combined patients with paroxysmal and persistent AF and analyzed them as one group. When looking at both groups separately (data not shown) no differences in peripheral blood pressures, PWV or PPA was seen between the study groups. There was lower central pulse pressure (36 mm Hg vs. 41 mm Hg, *p* = .020) and higher heart rate (63 vs. 56 bpm, *p* = .002) in the cardioversion group. Because the patient groups were relatively small, we combined the groups to maintain the statistical power of the study.

Metoprolol was the main beta blocker used in the study population. Because BBs have different affinities to beta receptors, then other drugs in the group could impact the central hemodynamic in a different manner.

## 5 CONCLUSIONS

Patients with AF have higher cSBP, cPP, PWV, and lower PPA compared to healthy patients, without differences in peripheral BP. These findings support the hypothesis that AS may play an important role in the development of AF. The use of BBs is related to the potential adverse effect on cBP, which may have an impact on the higher residual cardiovascular risk in patients with AF.

## CONFLICTS OF INTEREST

The authors declare no conflicts of interests.

## AUTHOR CONTRIBUTIONS

Priit Pauklin acquired data, interpreted the results, drafted and revised the manuscript, approved the final version. Jaan Eha interpreted the results, revised the manuscript, approved the final version. Kasper Tootsi interpreted the results, revised the manuscript, approved the final version. Rein Kolk interpreted the results, revised the manuscript, approved the final version. Rain Paju interpreted the results, revised the manuscript, approved the final version. Mart Kals interpreted the results, revised the manuscript, approved the final version. Pritt Kampus designed the work, drafted and revised the manuscript, approved the final version.
